# Publication Bias in Randomized Controlled Trials of Hypospadias Surgical Repair: A Systematic Review

**DOI:** 10.1111/iju.70153

**Published:** 2025-06-16

**Authors:** Ebram Nainggolan, Irfan Wahyudi, Arry Rodjani, Gerhard Reinaldi Situmorang, Muhammad E. H. Chowdhury, Putu Angga Risky Raharja, Tariq Abbas

**Affiliations:** ^1^ Department of Urology, Faculty of Medicine Universitas Indonesia—Cipto Mangunkusumo General Hospital Jakarta Indonesia; ^2^ College of Medicine, Qatar University Doha Qatar; ^3^ Urology Division, Department of Surgery Sidra Medicine Doha Qatar

**Keywords:** Egger's test, funnel plot, hypospadias, publication bias, randomized clinical trials

## Abstract

**Objectives:**

Hypospadias is a common congenital condition in males that has been subject to extensive research. However, publication bias can distort the scientific literature and impact clinical decision‐making. This review evaluates publication bias in hypospadias research and emphasizes the need for balanced reporting to improve clinical practices and enhance patient care.

**Methods:**

A literature search was conducted using four public databases to identify peer‐reviewed randomized controlled trials (RCT) of surgical interventions for hypospadias. The primary outcomes were success rate and complication rate. We assessed the publications using the Risk of Bias 2 (RoB‐2) tool and evaluated the quality of evidence using the GRADE method. Data were collated and recalculated using RevMan version 5.4. Publication bias was evaluated using a funnel plot and Egger's test.

**Results:**

The study included 32 articles for detailed analysis. The standard of evidence varied, with 8 studies rated as low quality, 23 as moderate, and 1 as high. Most studies lacked details on randomization, allocation concealment, and blinding, thus raising bias concerns. The funnel plot revealed a symmetrical distribution, and Egger's test indicated mild evidence of publication bias (*p* = 0.055).

**Conclusions:**

This study found no significant publication bias in hypospadias research, indicating a balanced evidence base at present. However, future studies should monitor bias through longitudinal assessments, explore new methodologies to mitigate bias, and increase visibility and accessibility of results to ensure the reliability of research findings.

## 1 Introduction

Hypospadias is a common birth defect in males, marked by the abnormal positioning of the urethral opening on the lower side of the penis [1]. Severity of this condition varies, with milder cases having the urethral opening near the tip of the penis, and more severe cases where it is positioned closer to the scrotum [2]. Incidence of hypospadias varies by region, with the highest rates in North America (34.2 cases per 10 000 births) and lowest prevalent in Asia (0.6 to 69 cases per 10 000 births). Given the frequency and complexity of hypospadias management, this disorder can place a significant strain on healthcare resources [3, 4, 5].

Research on hypospadias is complex and multifaceted, resulting in hundreds of papers being published every year on various aspects of this condition. Key areas of investigation include the genetic and environmental factors that predispose to hypospadias, advances in surgical techniques and their outcomes, postoperative care, and long‐term quality‐of‐life for patients. Each of these areas contributes to a comprehensive understanding of hypospadias and informs clinical practices that ultimately aim to improve patient outcomes [6]. High‐quality evidence enables clinicians to offer the best possible care, informed by the latest findings on the causes, management, and long‐term effects of hypospadias [5]. For research to significantly inform clinical practice, it is imperative that the evidence base is comprehensive and unbiased. This ensures that treatment recommendations are based on a full understanding of the condition and its management, rather than a skewed subset of studies that may over represent positive or significant findings [7].

Studies that yield positive or statistically significant results are more likely to be published compared to those with negative or inconclusive findings, a phenomenon known as publication bias [7]. This bias can arise from the preferences of researchers, reviewers, and journal editors, who may favor studies that report novel or favorable findings [8, 9]. Prior research has shown that studies with statistically significant results are more often published in high‐impact journals and tend to receive more citations, leading to overestimation of treatment effects and a skewed perception of risk factors [10, 11]. This distortion of the scientific literature can mislead clinicians, researchers, and policymakers, potentially resulting in suboptimal patient care and misguided research priorities [9]. Statistical methods can be used to identify these issues by assessing meta‐analyses for publication bias and inflated treatment effects [12]. Publication bias in hypospadias research could result in inflated success rates for certain surgical techniques or an exaggerated understanding of specific risk factors. This can affect clinical decisions, such as the choice of surgical method, and influence the direction of future research [8, 10].

This systematic review assessed the presence and magnitude of publication bias in randomized controlled trials (RCTs) focused on hypospadias surgical repair. By systematically reviewing and analyzing the available literature, this study aimed to discern patterns of bias and assess the impact on reported outcomes, thus providing valuable insight for clinicians, researchers, and policymakers. This review highlights the need for more transparent and balanced reporting of research findings, ultimately leading to more informed clinical decisions and better patient care.

## 2 Methods

### 2.1 Search Methodology and Inclusion Criteria

A comprehensive search of the public databases PubMed, CENTRAL, EBSCOhost, and Scopus was performed using relevant MeSH terms/keywords as shown in Table S1. There were no constraints on the publication date for the search. Reference lists from selected studies were examined to locate additional relevant articles. Two of the authors (P.A.R.R. and E.N.) carried out the screening process independently. Included were peer‐reviewed RCTs that reported on various surgical and non‐surgical interventions for patients with hypospadias, encompassing all age groups and demographic backgrounds. The primary outcomes considered were success rate and complication rate. Studies were excluded if they lacked full‐text availability, used animal models, were preliminary/pilot studies, or were written in languages other than English. A critical appraisal of the selected studies was performed according to guidelines from the Center for Evidence‐Based Medicine (Oxford University) [13].

### 2.2 Data Extraction and Statistical Analysis

The following data were collected: number of participants, age, intervention, comparison, and results [14]. The potential bias in each study was independently evaluated by two assessors, in line with the Cochrane Handbook for Systematic Reviews of Interventions [15]. Any differences in assessments were settled through discussion. The Risk of Bias 2 (RoB 2) tool was employed to evaluate the presence of bias [16]. Additionally, we assessed evidence quality using the Grading of Recommendations Assessment, Development and Evaluation (GRADE) method [17]. To evaluate publication bias among the included studies, we utilized two main approaches: visual inspection via a funnel plot and a statistical test for symmetry. First, we generated a funnel plot using RevMan version 5.4. This plot visually represents the potential for publication bias by displaying treatment effects from individual studies against a measure of study size or precision [18]. If no publication bias exists, the funnel plot should resemble a symmetrical, inverted funnel shape. Egger's test for funnel plot asymmetry was then conducted using the metafor package in R Studio version 4.2.3 [19]. This test evaluates the relationship between treatment effects and the studies' standard errors within the meta‐analysis. A significant *p*‐value from Egger's test (usually < 0.05) indicates possible asymmetry in the funnel plot, suggesting the presence of publication bias [17, 19]. Data on age at surgery and postoperative follow‐up were inconsistently reported across studies and therefore were not included in the final analysis.

## 3 Results

### 3.1 Study Characteristics

**FIGURE 1:**
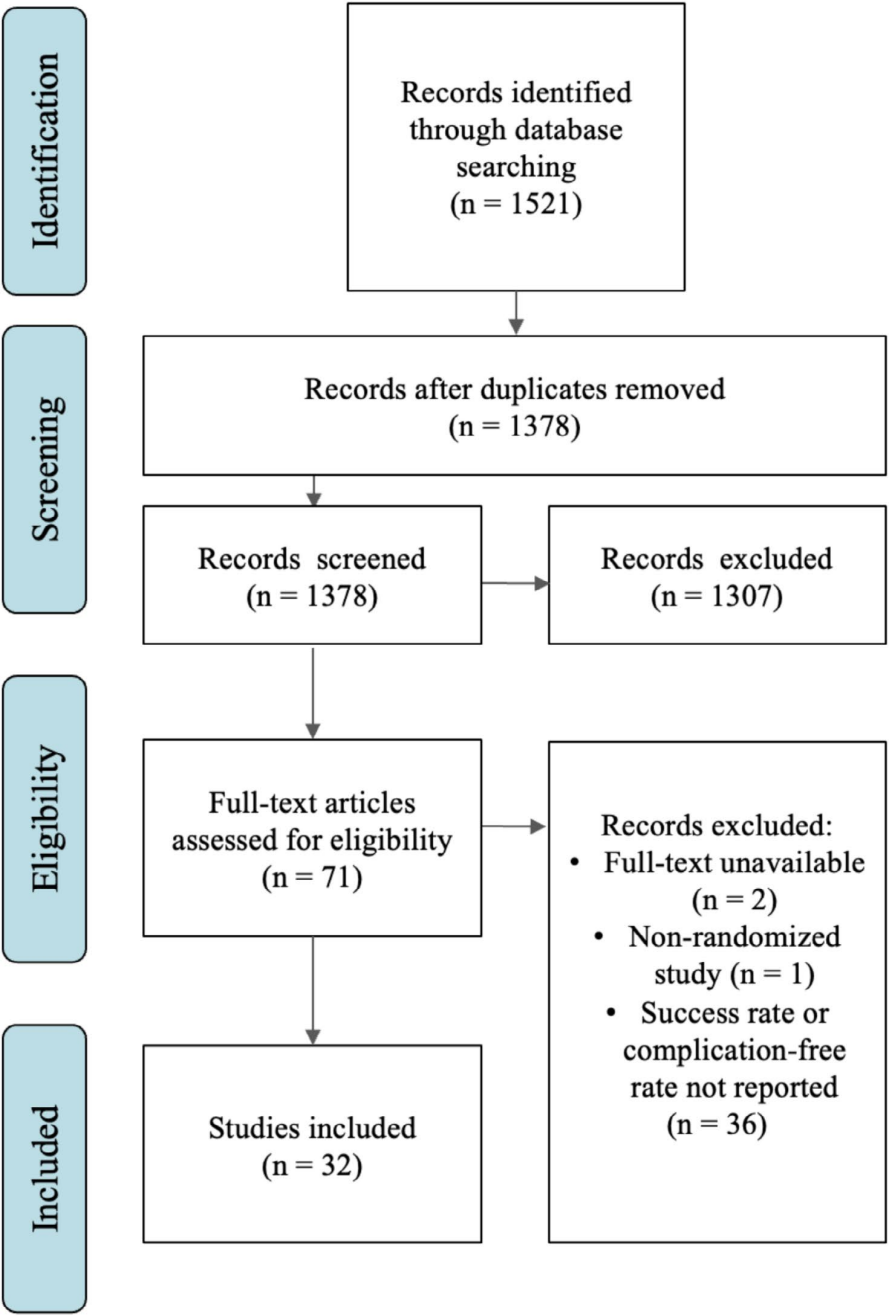
PRISMA diagram of the study selection process.

The initial search yielded 1521 total articles. After removing duplicates, 1378 articles were reviewed for relevance, and 71 articles underwent advanced screening. After eligibility assessment, we included 32 articles for detailed analysis. Figure 1 illustrates each step of the study selection process.

The current review comprised 32 RCTs representing a total sample size of 3425 participants across the included studies. In terms of participant categorization, five studies included patients with primary distal penile hypospadias, four studies focused on mid or proximal penile hypospadias, one study on penoscrotal hypospadias, two studies were on isolated anterior hypospadias, seven studies included various types of hypospadias (including anterior, distal, posterior, and mid‐penile), one study involved circumcised adult men undergoing cosmetic repair, and three studies included primary cases with minimal or no chordee. Regarding interventions, 18 studies compared different surgical techniques, five studies compared various flap methods (e.g., dorsal dartos flap vs. ventral dartos flap), and four studies compared additional procedures or materials (e.g., fibrin sealant usage, stented vs. unstented repair). Quality of evidence varied, with eight studies categorized as low quality, 23 as moderate quality, and one as high quality according to GRADE criteria. The primary factors contributing to the downgrading of evidence quality were concerns over bias and small sample size. A summary of the studies included for analysis is provided in Table S2.

### 3.2 Risk of Bias Among Included Studies

**FIGURE 2:**
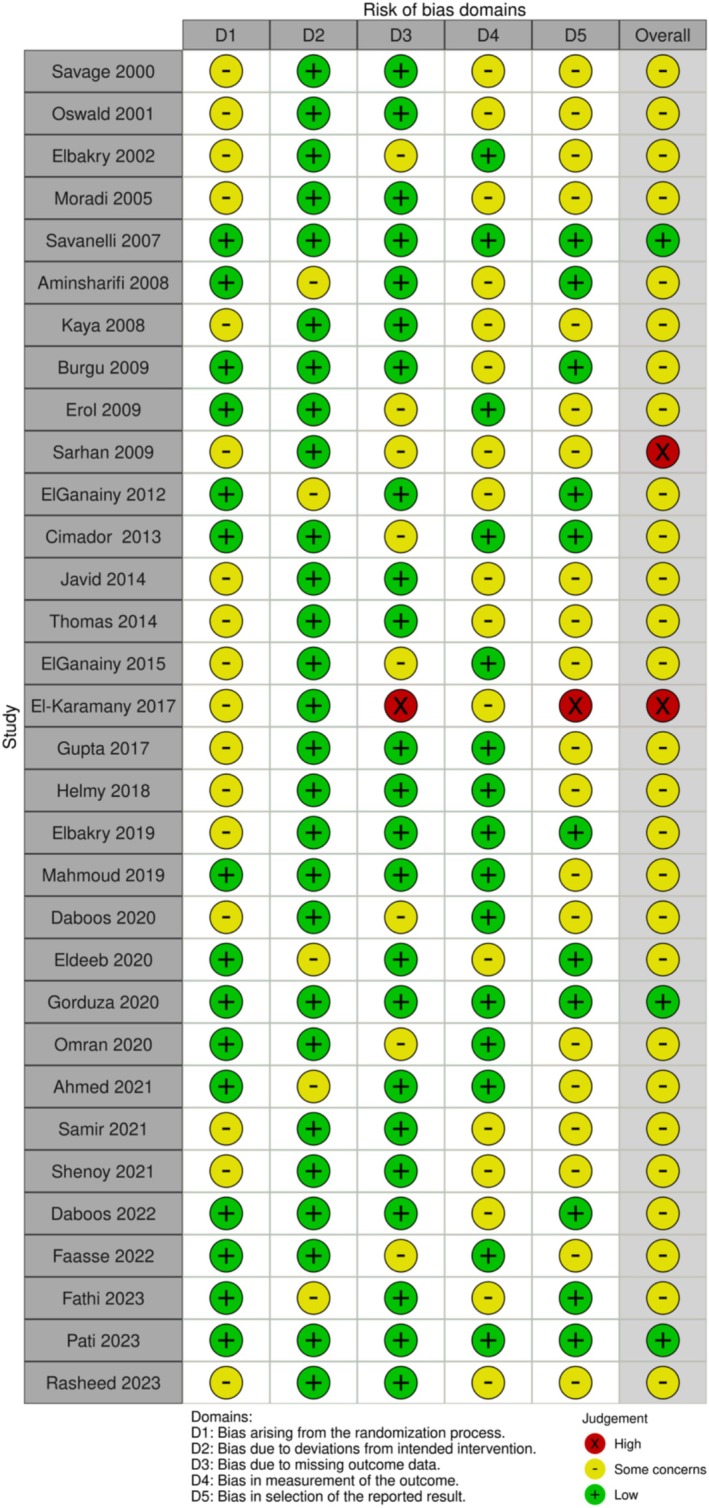
Risk of bias diagram.

**FIGURE 3:**
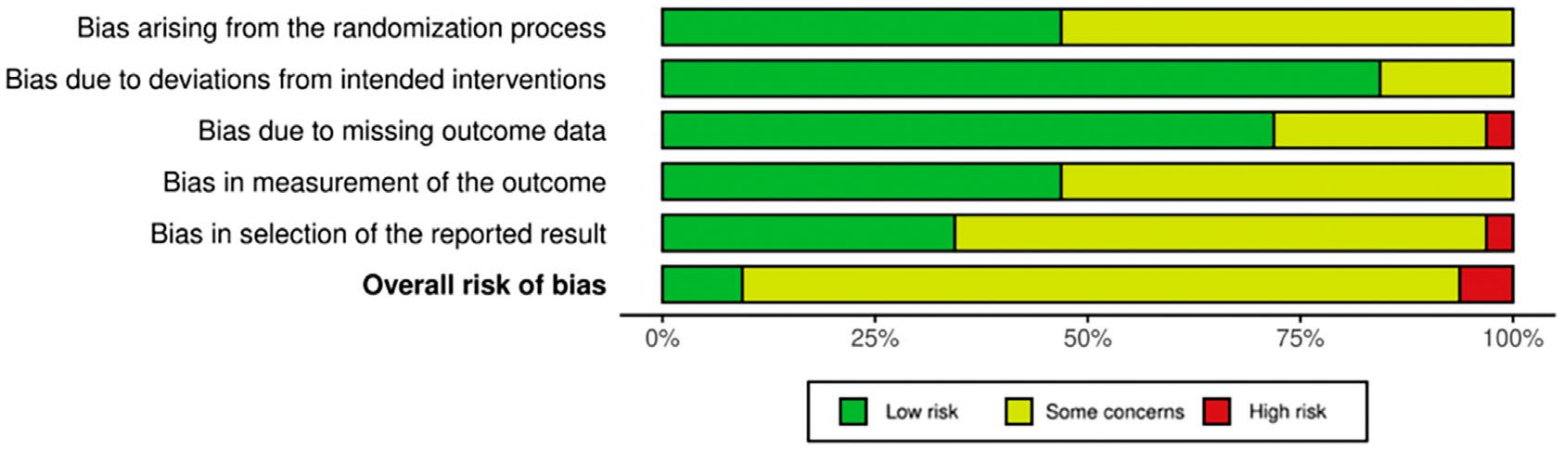
Risk of bias summary.

Across the included studies, the risk of bias generally varied from low to high, as depicted in Figures 2 and 3. Two included studies demonstrated a high overall risk of bias (El‐Karamany et al. and Sarhan et al.), representing 6.25% of the publications analyzed. Three studies displayed a low overall risk of bias (Gorduza et al., Pati et al., and Savanelli et al.), accounting for 9.375% of publications. The remaining 27 studies, making up 84.375% of the included publications, presented some concerns regarding bias (primarily due to a lack of evidence that pre‐specified analysis plans were finalized before outcome data became available). Moreover, most RCTs lacked sufficient details regarding the randomization process, allocation concealment, and whether outcome assessors were blinded to the intervention. The absence of this information raises concerns about potential bias in both the randomization process and measurement of outcomes.

### 3.3 Funnel Plot and Egger's Test

**FIGURE 4:**
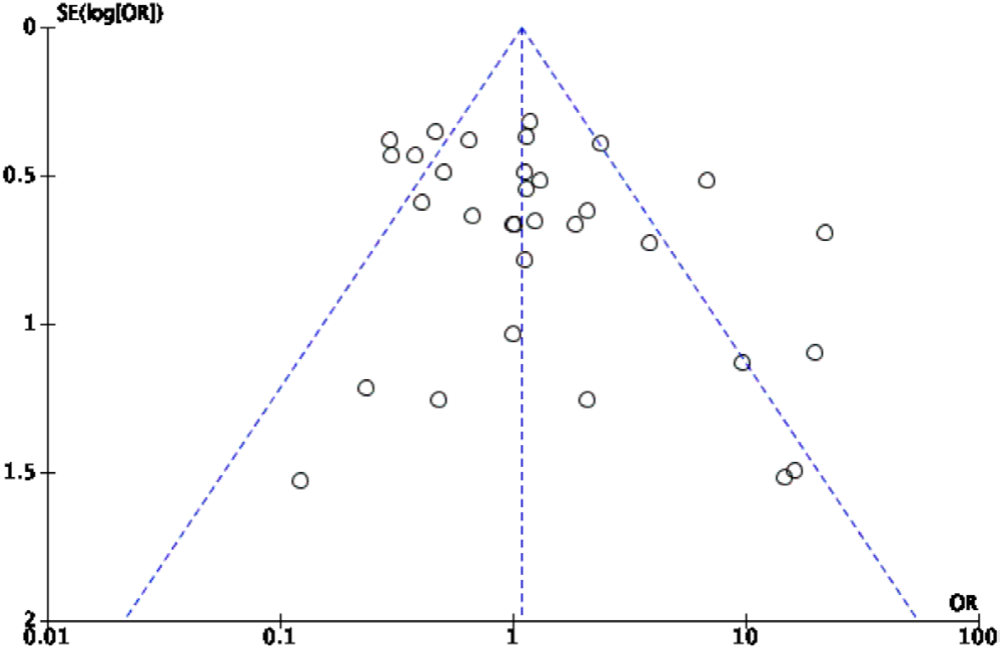
Funnel plot of 32 hypospadias studies.

Figure 4 depicts a funnel plot illustrating the 32 studies incorporated in our analysis. The plot showcases the odds ratio (OR) on the *x*‐axis (logarithmic scale), and the standard error (SE) of the log odds ratio on the *y*‐axis. Each circle within the plot corresponds to an individual study. A symmetrical distribution of studies around the overall effect estimate indicates a balanced spread of data points. Egger's test for publication bias produced a *p*‐value of 0.055, suggesting minimal evidence of publication bias in hypospadias research. The observed symmetry of the funnel plot also gives little indication of publication bias.

This systematic review found that a majority of RCTs in the hypospadias field showed a high risk of bias. Utilizing the ROB2 and GRADE tools, several key areas of concern were identified, including issues with randomization, blinding of participants and staff, and outcome reporting methods. These elements are crucial for ensuring accurate and reliable results, and when not properly addressed, trial quality may be compromised.

Bias is a common challenge in surgical trials, especially since it is often difficult to achieve proper blinding. For hypospadias repair techniques, it can be hard to blind participants and the healthcare providers to treatment‐related aspects, thus increasing risk of performance bias. Additionally, when researchers assessing patient outcomes are not blinded, detection bias can also occur, thereby influencing the accuracy of results obtained. These issues highlight the need for more meticulous trial designs to minimize bias and improve the reliability of surgical research findings.

## 4 Discussion

### 4.1 Key Findings

Publication bias is a well‐recognized challenge in the field of medical research. This phenomenon pertains to the tendency for studies reporting positive or significant findings to be published more readily in comparison to those yielding negative or non‐significant results.54 First identified as an issue over 50 years ago, numerous studies have since provided strong evidence of bias by tracking studies from protocol approval through to publication [20]. Problems arise when studies with null results are deemed unworthy of writing up and submission by researchers or when they receive less favorable treatment during peer review, making these less likely to undergo publication. Additionally, studies with statistically meaningful results have a greater probability of being accepted for publication in prestigious journals [12]. This bias can lead to a distorted understanding of the efficacy and safety of different medical interventions [21]. Since hypospadias is a prevalent congenital anomaly that has been widely studied and researched, assessing publication bias in this field is crucial given the potential impact on clinical decision‐making and treatment outcomes [7, 11, 21].

The key findings of our study indicate non‐significant publication bias in current hypospadias research, as evidenced by both funnel plot analysis and Egger's test results. The funnel plot indicates the expected range for 95% of studies, with symmetry around the central line suggesting no publication bias [22]. Several methodological studies using data from the Cochrane Database of Systematic Reviews (CDSR) have evaluated different tests for asymmetry, identifying Egger's linear regression as the most sensitive detection method [18]. In our analysis, the funnel plot displayed a symmetrical distribution of studies around the overall effect estimate, suggesting no significant publication bias among the included studies. Quantitative evaluation of the funnel plot using Egger's regression test generated a *p*‐value of 0.055, which is slightly above the typical threshold for significance. While this *p*‐value suggests a trend towards asymmetry, it does not offer substantial evidence of significant publication bias at present and instead highlights the importance of continued vigilance.

### 4.2 Comparison With Similar Research

Publication bias is prevalent in many medical disciplines. Egger et al. [23] identified asymmetry of funnel plots in 38% of meta‐analyses in prominent general medicine journals and 13% of reviews in the CDSR. Indeed, Sutton et al. [24] estimated that approximately half of *n* = 48 Cochrane reviews were missing studies. Similarly, another study using a selection model on 1106 meta‐analyses discovered that positive outcomes were 27% more likely to be included [25]. Schwab et al. [26] found that of 1054 meta‐analyses exhibiting small‐study effects, 214 (20%) displayed likely publication bias, representing 3.9% (95% CI 3.4% to 4.4%) of the total 5534 meta‐analyses examined. The intercepts from Egger's tests were more pronounced in meta‐analyses suspected of having publication bias. Conversely, a meta‐analysis by van Aert et al. [27] found no clear evidence of publication bias by Egger's test in 12.2% of subsets of 83 meta‐analyses from the CDSR, although statistically significant overestimation was noted. Our study revealed that hypospadias research exhibits low prevalence of publication bias, perhaps due to reduced competitive pressure and incentives for selective reporting in this specialized field. Although our findings suggest minimal publication bias, it is essential to consider the potential to overestimate the success of different surgical techniques, as well as the influence of specific environmental and genetic risk factors.

### 4.3 Explanations of Findings

Our analysis indicated that 27 of 32 RCTs (84.375%) presented some concerns regarding bias, primarily due to lack of information on the randomization method, allocation concealment, and blinding of outcome evaluators. Randomization is a core feature that distributes confounding factors evenly between groups and distinguishes RCTs from observational studies [27]. This process involves two key steps: generating a random number and concealing this number from the dispensing physician, which is key to preventing selection bias. It is also essential that independent outcome assessors are blinded in order to minimize detection bias. Trials that did not blind outcome assessors tended to report significantly greater treatment effects compared to those that did implement blinding. This raises concerns about the objectivity and impartiality of outcome measures, as assessors may unconsciously favor better results if aware of the exposure [28].

The credibility of a RCT is compromised in the absence of crucial methodological elements, as a failure to report these elements often signifies a failure to implement them [29]. The current analysis found that of 32 RCTs included, only 1 study had high‐quality evidence based on GRADE criteria (23 studies were rated as moderate quality, and 8 as low quality). The main factors downgrading evidence quality were the limited numbers of participants: our analysis included 3425 total patients with a mean of 107 per study. A review by Braga et al. [29] found that hypospadias trials generally have a limited number of participants, with an average sample size of 77 patients. Furthermore, sample size justification was not reported in almost all the included RCTs. Having an adequate number of participants in an RCT is essential to achieve the necessary statistical power to identify genuine effects while minimizing the likelihood of type II error. Larger sample sizes also provide more precise effect estimates with narrower confidence intervals, thus enhancing the reliability of results and improving external validity/applicability to a broader population. Adequately powered studies are also ethically imperative, as they justify exposing participants to interventions by ensuring the study can yield meaningful results [30].

Adequately powered studies are also ethically imperative, as they justify exposing participants to interventions by ensuring the study can yield meaningful results. In addition to conventional clinical and procedural factors, variables such as the type of suture material, perioperative antibiotic protocols, use of tissue healing agents, dressing techniques, surgeon specialty, use of magnification, and country‐specific practices can also contribute to outcome variability and potential bias. Future studies should consider harmonizing these factors or clearly reporting them to enhance comparability and reduce residual confounding.

### 4.4 Strengths, Limitations and Actions Needed

Despite the high risk of publication bias identified by ROB2 and GRADE assessments, analysis of the funnel plot and Egger's test suggested this was unlikely to be significant at present. The *p*‐value of 0.055 derived from Egger's test does not definitively eliminate the possibility of publication bias, but it does provide some reassurance regarding overall integrity of the included studies. The results observed across the included trials therefore do not appear to have been heavily influenced by selective reporting or underreporting of negative outcomes, but transparent reporting and publication practices will nonetheless be key to maintaining a balanced evidence base going forwards. Encouraging publication of negative or inconclusive results will help achieve this goal. Researchers should adhere to rigorous methodological standards, consider pre‐registering studies, and utilize comprehensive databases and registries to ensure all research findings are accessible. These practices will contribute to a more accurate and reliable body of evidence in future.

In summary, while significant methodological concerns were detected in the RCTs analyzed here, current statistical evidence of publication bias appears weak, as indicated by symmetrical funnel plot and a non‐significant Egger's test. This finding nonetheless emphasizes the need for meticulous trial design and improved reporting standards in future hypospadias research. Addressing methodological issues, such as randomization processes, participant and personnel blinding, and outcome reporting, will be crucial to enhancing the credibility of surgical trials in this field. Maintaining transparency and encouraging publication of all results, regardless of outcome, will also sustain a more balanced and accurate evidence base.

The current results also highlight the importance of interpreting meta‐analytical findings with caution. While statistical bias measures such as funnel plots and Egger's test can provide useful insights, they must be considered alongside the quality of the underlying evidence. Ensuring that both the design and reporting of hypospadias trials meet higher standards will not only improve individual study outcomes, but also strengthen the overall conclusions drawn from meta‐analyses.

Future studies should continue to monitor publication bias in hypospadias research through longitudinal assessments to detect any changes over time. Exploring new methodologies or technologies to detect and mitigate publication bias can further enhance the reliability of study findings. In addition to rigorous trial design, researchers should also focus on increasing standards of outcome reporting, such as visibility and accessibility of all research results, which will contribute to a comprehensive and unbiased understanding of hypospadias and its treatments. Particular attention should be given to consistently reporting key clinical variables, such as age at surgery and duration of postoperative follow‐up, which were frequently missing in the current literature and could not be analyzed in our review. Standardized reporting of these factors will allow for more meaningful comparisons across studies and improve the quality of future meta‐analyses.

## Author Contributions

**Ebram Nainggolan:** data curation, writing – original draft. **Irfan Wahyudi:** writing – original draft, investigation, data curation. **Arry Rodjani:** investigation, methodology, data curation, supervision. **Gerhard Reinaldi Situmorang:** supervision, resources, visualization, writing – original draft. **Muhammad E. H. Chowdhury:** resources, investigation. **Putu Angga Risky Raharja:** conceptualization, methodology, data curation, investigation, validation, supervision, visualization, project administration, writing – review and editing. **Tariq Abbas:** conceptualization, methodology, supervision, validation, investigation, visualization, writing – review and editing.

## Ethics Statement

The authors have nothing to report.

## Consent

The authors have nothing to report.

## Conflicts of Interest

The authors declare no conflicts of interest.

## Supporting information


**Table S1.** Search Strategy.
**Table S2.** Summary of study results.
