# Supplementary material for: Publication Bias in Randomized Controlled Trials of Hypospadias Surgical Repair: A Systematic Review
Source: Int J Urol. 2025 Jun 16;32(10):1405–12. doi: 10.1111/iju.70153 (PMC12503199; doi:10.1111/iju.70153)
Supplement: Supplementary file 1 — Table S1. Search Strategy. Table S2. Summary of study results. [file IJU-32-1405-s001.docx]

**Supplementary Table 1.** Search strategy

| **Database [date of search]** | **Search strategy** | **Hits** |
| --- | --- | --- |
| PubMed [2024 July 7] | "Hypospadias"[Mesh]  Limit to: full-text, randomized controlled trials | 108 |
| CENTRAL [2024 July 7] | MeSH descriptor: [Hypospadias] explode all trees  Limit to: English | 215 |
| Scopus [2024 July 7] | ( hypospadias ) AND ( trial* ) AND ( LIMIT-TO ( SRCTYPE , "j" ) ) AND ( LIMIT-TO ( DOCTYPE , "ar" ) ) AND ( LIMIT-TO ( SUBJAREA , "MEDI" ) ) AND ( LIMIT-TO ( LANGUAGE , "English" ) ) AND ( LIMIT-TO ( PUBSTAGE , "final" ) ) AND ( LIMIT-TO ( EXACTKEYWORD , "Controlled Study" ) ) | 736 |
| EBSCOhost [2024 July 7] | (MM "Hypospadias")  Limit to: full-text, peer reviewed, English | 462 |

**Supplementary Table 2.** Summary of study results

| **Author (year)** | **Participants** | **N** | **Ages** | **Intervention**  **(Group 1)** | **Comparison**  **(Group 2)** | **Results** | **QoE (GRADE)** |
| --- | --- | --- | --- | --- | --- | --- | --- |
| Ahmed et al. (2021)^1^ | Patients with primary distal penile hypospadias | 107 | Mean: 33.6 ± 39.4 months in group 1 and 23.1 ± 14.6 months in group 2 | G-TIP | C-TIP | Success rate of 90.7% (49/54) in group 1 and 90.5% (48/53) in group 2 (OR: 1.021; SE: 0.664) | Moderate |
| Aminsharifi et al. (2008)^2^ | Patients with primary distal hypospadias | 40 | Mean: 7.05 ± 4.85 years in group A and 7.31 ± 2.17 years in group B | Mathieu–Incised Plate technique (Mathieu-IP) | Snodgrass urethroplasty | Complication free rate of 100% (20/20) in group 1 and 75% (15/20) in group 2 (OR: 1.333; SE: 0.465) | Moderate |
| Burgu et al. (2009)^3^ | Circumcised adult men who underwent primary distal or mid-penile hypospadias repair for cosmetic reasons | 42 | Mean: 21.8 (20-26) years | TIPU with dorsal dartos flap | TIPU with ventral dartos flap | Success rate of 82% (18/22) in group 1 and 80% (16/20) in group 2 (OR: 1.125; SE: 0.786) | Moderate |
| Cimador et al. (2013)^4^ | Patients with isolated anterior hypospadias, first urethral repair, no preoperative topical androgen therapy, TIPU procedure according to Snodgrass | 130 | 16-36 months | Dorsal preputial flap | Ventral dartos flap | Success rate of 63.2% (36/57) in group 1 and of 72.6% (53/73) in group 2 (OR: 0.647; SE: 0.380) | Moderate |
| Daboos et al. (2020)^5^ | Patients with peno-scrotal hypospadias | 160 | Mean: 4.2 years in group 1 and 4.5 years in group 2 | Double-faced tubularized preputial flap technique | Standard ventral preputial tubularized flap technique | Complication free rate of 85% (62/80) in group 1 and 75% (60/80) in group 2 (OR: 1.889; SE: 0.406) | Low |
| Daboos et al. (2022)^6^ | Patients with anterior, mid-penile, or posterior penile hypospadias, with shallow and narrow urethral plate size < 6 mm, who underwent single-stage repair | 68 | Mean: 3.2 years in group 1 and 3.3 years in group 2 | Double-faced transverse preputial onlay island flap | Inner transverse preputial onlay island flap | Complication free rate of 85.3% (29/34) in group 1 and 73.6% (25/34) in group 2 (OR: 2.088; SE: 0.621) | Moderate |
| Elbakry et al. (2002)^7^ | Patients who underwent a TIP repair of hypospadias | 64 | Mean: 7.2 years, range 2 ± 18 | Regular neourethral dilatation | No neourethral dilatation | Success rate of 97.4% (37/38) in group 1 and 65.4% (17/26) in group 2 (OR: 19.588; SE: 1.094) | Moderate |
| Elbakry et al. (2019)^8^ | Patients with distal penile, coronal, or glanular hypospadias with a moderately grooved urethral plate of 8–10 mm width, according to the Holland and Smith classification | 349 | Mean: 2.8 (1.09, 1–5) years in group 1 and 2.5 (1.07, 1–5) in group 2 | TIPU | Tubularisation of the intact plate with lateral  augmentation of the urethral plate using penile skin | Success rate of 83.2% (143/172) in group 1 and 94.4% (167/177) in group 2 (OR: 0.295; SE: 0.384) | Moderate |
| Eldeeb et al. (2020)^9^ | Non-circumcised patients with primary distal penile hypospadias | 60 | 4-24 months | TIPU | TIPU with preputial graft (Snodgraft) | Complication free rate of 93.3% (28/30) in group 1 and 93.3% (28/30) in group 2 (OR: 1; SE: 1.035) | Low |
| ElGanainy et al. (2012)^10^ | Patients with primary coronal or distal penile hypospadias, and with normal-sized urethral meatus, healthy ventral penile skin (without chordee or previous penile surgery) | 153 | Mean: 35.2 (8-78) months in group I and 35.2 (9-76) months in group II | Parameatal-based skin flap (Mathieu urethroplasty) with prepuce preservation (without penile degloving) | Parameatal-based skin flap with penile degloving, dartos layer cover, midline incision of the preputial skin with creation of Bayer’s flaps for ventral skin coverage, and excision of the rest of the prepuce | Complication free rate of 90.7% (78/86) in group 1 and 89.5% (60/67) in group 2 (OR: 1.138; SE: 0.545) | Moderate |
| ElGanainy et al. (2015)^11^ | Patients with coronal, subcoronal, or distal penile hypospadias, with a urethral plate width of 66 mm, and minimal or no chordee | 60 | Mean: 40.7 (17.3, 9–76) months in group 1 and 41.1 (18.9, 14–83) in group 2 | MOIF using a midline longitudinal outer preputial skin flap passed ventrally by penile button-holing through dartos fascia incision | Mathieu urethroplasty | Complication free rate of 100% (30/30) in group 1 and 80% (24/30) in group 2 (OR: 16.184; SE: 1.483) | Low |
| El-Karamany et al. (2017)^12^ | Patients with distal hypospadias | 93 | Mean: 3 (0.5-10) years in group 1 and 2.5 (0.5-12) in group 2 | Stented TIPU | Unstented TIPU | Success rate of 91% (42/46) in group 1 and 85% (40/47) in group 2 (OR: 1.838; SE: 0.665) | Low |
| Erol et al. (2009)^13^ | Patients with primary coronal or subcoronal hypospadias | 77 | Mean: 29.4±17.4 (9–72) months in group 1 and 28.8±15.9 (9–71) months in group 2 | Monolayer dartos coverage | Double-layer dartos coverage | Complication free rate of 91.9% (34/37) in group 1 and 100% (40/40) in group 2 (OR: 0.122; SE: 0.529) | Moderate |
| Faasse et al. (2022)^14^ | Boys with a primary, single-stage repair of mid-to-distal hypospadias with placement of an open-drainage urethral stent | 93 | Mean: 10 (8-12) months | Trimethoprim- sulfamethoxazole twice daily for 10 days postoperatively | Placebo twice daily for 10 days postoperatively | Complication free rate of 78% (35/45) in group 1 and 90% (43/48) in group 2 (OR: 0.407; SE: 0.593) | Moderate |
| Fathi et al. (2023)^15^ | Patients with primary hypospadias and absence of severe chordee | 50 | Mean: 4.2 years | Urethral advancement and glanuloplasty | TIPU | Complication free rate of 68% (17/25) in group 1 and 76% (19/25) group 2 (OR: 0.671; SE: 0.635) | Low |
| Gorduza et al. (2020)^16^ | Patients with hypospadias associated with a midshaft or more proximal division of the corpus spongiosum who underwent transverse island flap urethroplasty | 241 | Mean: 15.1 (±4.8) months in group 1 and 14.9 (±5.1) in group 2 | Application of promestriene 1% for 2 months before surgery | Application of placebo gel for 2 months before surgery | Complication free rate of 80.7% (96/119) in group 1 and 77.9% (95/122) in group 2 (OR: 0.186; SE: 0.319) | High |
| Gupta et al. (2017)^17^ | Patients with primary subcoronal, distal- or mid-penile hypospadias | 100 | Mean: 26.2 (12–66) months | Interrupted subcuticular suture Snodgrass TIP urethroplasty | Continuous subcuticular suture Snodgrass TIP urethroplasty | Complication free rate of 80% (40/50) in group 1 and 78% (39/50) in group 2 (OR: 1.128; SE: 0.491) | Moderate |
| Helmy et al. (2018)^18^ | Patients with distal hypospadias (sub-coronal or distal shaft), primary, uncircumcised, and no or mild chordee (<30°) | 60 | Mean: 40 ± 15 months | TIPU | G-TIP | Success rate of 96.7% (29/30) in group 1 and 93.3% (28/30) in group 2 (OR: 1.036; SE: 1.440) | Moderate |
| Javid et al. (2014)^19^ | Patients with mid or proximal penile hypospadias | 60 | Mean: 3±0.65 years in group I and 2±0.45 years in group II | Urethroplasty | Flap repair | Complication free rate of 90% (27/30) in group 1 and 70% (21/30) in group 2 (OR: 3.857; SE: 0.727) | Low |
| Kaya et al. (2008)^20^ | Patients with primary hypospadias | 75 | Mean: 33.4 ±3.7 months | Application of 2.5% transdermal gel daily | No application of 2.5% transdermal gel daily | Complication free rate of 91.9% (34/37) in group 1 and 73.6% (13/38) in group 2 (OR: 21.79; SE: 0.693) | Moderate |
| Mahmoud et al. (2019)^21^ | Patients with primary subcoronal, distal or mid-penile hypospadias and minimal chordee | 180 | 12-65 months | TIPU with platelet-rich plasma coverage layer | TIPU with ventral dartos flap | Success rate of 87% (78/90) in group 1 and 73% (66/90) in group 2 (OR: 2.363; SE: 0,391) | Moderate |
| Moradi et al. (2005)^22^ | Patients with anterior distal shaft hypospadias | 33 | Mean: 7.06 ± 3.44 (range 2 to 12) | Snodgrass technique | Mathieu technique | Success rate of 80.02% (12/15) in group 1 and 94.45% (17/18) in group 2 (OR: 0.286; SE: 1.22) | Moderate |
| Omran et al. (2020)^23^ | Patients with primary anterior or middle hypospadias with narrow urethral plates | 39 | 9-32 months in group 1 and 8-34 months in group 2 | DIGU | LOF | Complication free rate of 94.7% (18/19) in group 1 and 65% (13/20) in group 2 (OR: 9.692; SE: 1.129) | Low |
| Oswald et al. (2001)^24^ | Patients undergoing primary distal hypospadias repair | 60 | Mean: 24.9 months, range 9±72 in group 1 and 23.1 months, range 7±19 in group 2 | Mathieu repair | Snodgrass procedure | Complication free rate of 93.3% (28/30) in group 1 and 96.7% (29/30) in group 2 (OR: 0.483; SE: 1.253) | Moderate |
| Pati et al. (2023)^25^ | Patients with proximal hypospadias requiring two-stage repairs (chordee correction followed by urethral tubularization) | 60 | Mean: 5.76±2.79 years in group 1 and 5.68±3.49 in group 2 | Application of topical estrogen cream (0.5 mg estriol) to the ventral penis for a month | Application of normal saline gel | Complication free rate of 55.2% (16/29) in group 1 and 48.4% (15/31) in group 2 (OR: 1.313; SE: 0.518) | Moderate |
| Rasheed et al. (2023)^26^ | Male pediatric patients (6 months- 12 years) with primary hypospadias, good prepuce, and wide urethral plate | 120 | Mean: 3.5±2.3 years | Bracka’s technique | Snodgrass technique | Complication free rate of 60% (36/60) in group 1 and 83.3% (50/60) in group 2 (OR: 0.3; SE: 0.435) | Moderate |
| Samir et al. (2021)^27^ | Uncircumcised hypospadiac boys with adequate urethral plate who underwent TIP repair | 229 | Median: 27 (22-31) months in group 1 and 26 (22-32) in group 2 | TIP repair using continuous subcuticular suture urethroplasty | TIP repair using interrupted subcuticular suture urethroplasty | Complication free rate of 75.9% (85/112) in group 1 and 87.2% (102/117) in group 2 (OR: 0.463; SE: 0.354) | Moderate |
| Sarhan et al. (2009)^28^ | Patients with primary anterior or mid-penile hypospadias | 80 | Mean: 4.5 years (range 3 to 7) | Continuous suture | Interrupted suture | Complication free rate of 87.5% (35/40) in group 1 and 85% (34/40) in group 2 (OR: 1.235; SE: 0.652) | Low |
| Savage et al. (2000)^29^ | Children with hypospadias | 98 | 0-18 years old | Dressing using a transparent, gas-permeable, waterproof, hypoallergenic, non-latex sterile film | No dressing | Success rate of 90% (44/49) in group 1 and 90% (44/49) in group 2 (OR: 1; SE: 0.667) | Moderate |
| Savanelli et al. (2007)^30^ | Patients with inferior hypospadias who underwent TIPU | 130 | 9 months to 12 years (median 26.9 months) | Non-covered urethroplasty | Covered urethroplasty (CU) | 44/65 complication-free patients in group 1 and 55/65 in group 2 (OR 0.382; SE 0.434) | Moderate |
| Shenoy et al. (2021)^31^ | Patients with hypospadias, irrespective of type | 100 | Mean: 4.6 years | Fibrin sealant usage to reinforce the urethroplasty | Routine hypospadias repair | Complication free rate of 88% (44/50) in group 1 and 52% (26/50) in group 2 (OR: 6.769; SE: 0.519) | Moderate |
| Thomas et al. (2014)^32^ | Patients undergoing TIPU repair for isolated anterior hypospadias | 214 | Mean: 42.7 months (median: 32 months, range: 5 months - 15 years) | TIPU repair with dorsal dartos flap | TIPU repair without dorsal dartos flap | Complication free rate of 87.9% (94/107) in group 1 and 93.5% (100/107) in group 2 (OR: 0.522; SE: 0.490) | Moderate |

*Abbreviation: QoE, quality of evidence; TIPU, tubularized incised plate urethroplasty; G-TIP, grafted tubularized incised plate; C-TIP classic tubularized incised plate; MOIF, modified onlay island flap; DIGU, dorsal inlay graft urethroplasty; LOF, longitudinal onlay preputial flap*

**Supplementary References:**

1. Ahmed S, Noureldin YA, Sherif H, Zahran A, Omar R. Cosmetic outcomes of grafted tubularized incised plate urethroplasty in primary distal penile hypospadias: prospective comparative study with the classic Snodgrass repair. African Journal of Urology. 2021 Dec 1;27(1). https://doi.org/10.1186/S12301-021-00255-6

2. Aminsharifi A, Taddayun A, Assadolahpoor A, Khezri A. Combined Use of Mathieu Procedure with Plate Incision for Hypospadias Repair: A Randomized Clinical Trial. Urology. 2008 Aug;72(2):305–8. https://doi.org/10.1016/J.UROLOGY.2008.02.034

3. Burgu B, Aydogdu O, Söylemez H, Soygur T. Both dorsal and ventral flaps can be used in previously circumcised hypospadic adults with comparable success rates. Int Urol Nephrol. 2010 Sep;42(3):689–95. https://doi.org/10.1007/S11255-009-9638-6

4. Cimador M, Pensabene M, Sergio M, Catalano P, de Grazia E. Coverage of urethroplasty in pediatric hypospadias: randomized comparison between different flaps. Int J Urol [Internet]. 2013 Oct [cited 2025 Jan 8];20(10):1000–5. Available from: https://pubmed.ncbi.nlm.nih.gov/23421595/ doi: 10.1111/IJU.12092

5. Daboos M, Helal AA, Salama A. Five years’ experience of double faced tubularized preputial flap for penoscrotal hypospadias repair in pediatrics. J Pediatr Urol [Internet]. 2020 Oct 1 [cited 2025 Jan 8];16(5):673.e1-673.e7. Available from: https://pubmed.ncbi.nlm.nih.gov/32800482/ doi: 10.1016/J.JPUROL.2020.07.037

6. Daboos M, Hefney K, Abdelhafez Mahmoud M, Salama A, Mohammed Y, Hussein M, et al. Evaluation of double faced transverse preputial (onlay) island flap for hypospadias repair in pediatrics: a randomized controlled study. Int Urol Nephrol. 2022 Jul 1;54(7):1471–7. https://doi.org/10.1007/S11255-022-03217-1

7. Elbakry A. Further experience with the tubularized-incised urethral plate technique for hypospadias repair. BJU Int [Internet]. 2002 [cited 2025 Jan 8];89(3):291–4. Available from: https://pubmed.ncbi.nlm.nih.gov/11856113/ doi: 10.1046/J.1464-4096.2001.01525.X

8. Elbakry A, Hegazy M, Matar A, Zakaria A. Tubularised incised-plate versus tubularisation of an intact and laterally augmented plate for hypospadias repair: A prospective randomised study. Arab J Urol. 2016 Jun 1;14(2):163–70. https://doi.org/10.1016/J.AJU.2016.03.004

9. Eldeeb M, Nagla S, Abou-Farha M, Hassan A. Snodgrass vs Snodgraft operation to repair the distal hypospadias in the narrow urethral plate. J Pediatr Urol [Internet]. 2020 Apr 1 [cited 2025 Jan 8];16(2):165.e1-165.e8. Available from: https://pubmed.ncbi.nlm.nih.gov/32144015/ doi: 10.1016/J.JPUROL.2020.01.006

10. Elganainy EO, Hameed DA, Abdelsalam YM, Abdelaziz MA. Prepuce preserving versus conventional Mathieu urethroplasty for distal hypospadias - A prospective randomized study. J Pediatr Urol. 2012;8(3):264–7. https://doi.org/10.1016/J.JPUROL.2011.05.004

11. ElGanainy EO. A modified onlay island flap vs. Mathieu urethroplasty for distal hypospadias repair: A prospective randomised study. Arab J Urol. 2015 Sep 1;13(3):169–75. https://doi.org/10.1016/J.AJU.2015.06.005

12. El-Karamany TM, Al-Adl AM, Omar RG, Abdel Aal AM, Eldakhakhny AS, Abdelbaki SA. A Critical Analysis of Stented and Unstented Tubularized Incised Plate Urethroplasty Through a Prospective Randomized Study and Assessment of Factors Influencing the Functional and Cosmetic Outcomes. Urology. 2017 Sep 1;107:202–8. https://doi.org/10.1016/J.UROLOGY.2017.04.056

13. Erol A, Kayikci A, Memik O, Cam K, Akman Y. Single vs. double dartos interposition flaps in preventing urethrocutaneous fistula after tubularized incised plate urethroplasty in primary distal hypospadias: a prospective randomized study. Urol Int [Internet]. 2009 Oct [cited 2025 Jan 8];83(3):354–8. Available from: https://pubmed.ncbi.nlm.nih.gov/19829040/ doi: 10.1159/000241682

14. Faasse MA, Farhat WA, Rosoklija I, Shannon R, Odeh RI, Yoshiba GM, et al. Randomized trial of prophylactic antibiotics vs. placebo after midshaft-to-distal hypospadias repair: the PROPHY Study. J Pediatr Urol. 2022 Apr 1;18(2):171–7. https://doi.org/10.1016/J.JPUROL.2022.01.008

15. Fathi BA, Elgammal AA, Ghoneimy OM, Alrefaey AA, Abouelgreed TA, Elhelaly MA, et al. Urethral advancement and glanuloplasty versus tubularized incised plate urethroplasty for distal hypospadias repair: a prospective randomized study. BMC Urol [Internet]. 2023 Dec 1 [cited 2025 Jan 8];23(1):1–9. Available from: https://bmcurol.biomedcentral.com/articles/10.1186/s12894-023-01242-5 doi: 10.1186/S12894-023-01242-5/TABLES/1

16. Gorduza D, Plotton I, Remontet L, Gay CL, Jani M El, Cheikhelard A, et al. Preoperative topical estrogen treatment vs placebo in 244 children with midshaft and posterior hypospadias results of a prospective, multicenter, double-blind, randomized, placebo-controlled trial. Journal of Clinical Endocrinology and Metabolism. 2020 Jul 1;105(7). https://doi.org/10.1210/CLINEM/DGAA231

17. Gupta A, Gupta R, Srivastav P, Gupta A. Comparison of interrupted- and continuous-suture urethroplasty in tubularised incised-plate hypospadias repair: A prospective study. Arab J Urol. 2017 Dec 1;15(4):312–8. https://doi.org/10.1016/J.AJU.2017.10.004

18. Helmy TE, Ghanem W, Orban H, Omar H, El-Kenawy M, Hafez AT, et al. Does grafted tubularized incided plate improve the outcome after repair of primary distal hypospadias: A prospective randomized study? J Pediatr Surg. 2018 Aug 1;53(8):1461–3. https://doi.org/10.1016/J.JPEDSURG.2018.03.019

19. Javid L, Pansota MS, Ahmad I, Tariq M, Tabassum SA. Comparison between tubularised incised plate urethroplasty and onlay island flap repair in mid and proximal penile hypospadias. J Pak Med Assoc. 2014 Apr;64(4):415–8.

20. Kaya C, Bektic J, Radmayr C, Schwentner C, Bartsch G, Oswald J. The efficacy of dihydrotestosterone transdermal gel before primary hypospadias surgery: A prospective, controlled, randomized study. Journal of Urology. 2008;179(2):684–8. https://doi.org/10.1016/J.JURO.2007.09.098

21. Mahmoud AY, Gouda S, Gamaan I, Baky Fahmy MA. Autologous platelet-rich plasma covering urethroplasty versus dartos flap in distal hypospadias repair: A prospective randomized study. Int J Urol [Internet]. 2019 Apr 1 [cited 2025 Jan 8];26(4):475–80. Available from: https://pubmed.ncbi.nlm.nih.gov/30719774/ doi: 10.1111/IJU.13912

22. Moradi M, Moradi A, Ghaderpanah F. Comparison of snodgrass and mathieu surgical techniques in anterior distal shaft hypospadias repair. Urol J. 2005;2(1):28–31.

23. Omran M, Sakr A, Elgalaly H, Fawzy A, Abdalla M. Narrow urethral plate augmentation in anterior and middle hypospadias repair: Onlay flap VS. Inlay graft. A prospective randomized comparative study. J Pediatr Urol. 2021 Apr 1;17(2):216.e1-216.e8. https://doi.org/10.1016/J.JPUROL.2020.11.026

24. Oswald J, Körner I, Riccabona M. Comparison of the perimeatal-based flap (Mathieu) and the tubularized incised-plate urethroplasty (Snodgrass) in primary distal hypospadias. BJU Int [Internet]. 2000 [cited 2025 Jan 8];85(6):725–7. Available from: https://pubmed.ncbi.nlm.nih.gov/10759674/ doi: 10.1046/J.1464-410X.2000.00479.X

25. Pati AB, Mishra P, Mahalik SK, Tripathy BB, Mohanty MK. Effect of preoperative estrogen on complications after proximal hypospadias repair: A randomized controlled trial. Indian Journal of Urology. 2023 Apr 1;39(2):126–32. https://doi.org/10.4103/IJU.IJU_387_22

26. Rasheed S, Fazlani R, Shaikh S, Irshad S, Khan MP, Memon S. Bracka’s and Snodgrass Surgical Techniques for Hypospadias Correction: A Comparative Study. Medical Forum Monthly [Internet]. 2023 Jan 30 [cited 2025 Jan 8];34(1):73–7. Available from: https://medicalforummonthly.com/index.php/mfm/article/view/73

27. Samir M, Mahmoud MA, Azazy S, Tawfick A. Does the suturing technique (continuous versus interrupted) have an impact on the outcome of tubularized incised plate in hypospadias repair with adequate urethral plate? A prospective randomized study. J Pediatr Urol. 2021 Aug 1;17(4):519.e1-519.e7. https://doi.org/10.1016/J.JPUROL.2021.04.021

28. Sarhan O, Saad M, Helmy T, Hafez A. Effect of Suturing Technique and Urethral Plate Characteristics on Complication Rate Following Hypospadias Repair: A Prospective Randomized Study. Journal of Urology. 2009 Aug;182(2):682–6. https://doi.org/10.1016/J.JURO.2009.04.034

29. Van Savage JG, Palanca LG, Slaughenhoupt BL. A prospective randomized trial of dressings versus no dressings for hypospadias repair. Journal of Urology. 2000;164(3 II):981–3. https://doi.org/10.1097/00005392-200009020-00015

30. Savanelli A, Esposito C, Settimi A. A prospective randomized comparative study on the use of ventral subcutaneous flap to prevent fistulas in the Snodgrass repair for distal hypospadias. World J Urol [Internet]. 2007 Dec [cited 2025 Jan 8];25(6):641–5. Available from: https://pubmed.ncbi.nlm.nih.gov/17912528/ doi: 10.1007/S00345-007-0215-2

31. Shenoy NS, Tiwari C, Gandhi S, Kumbhar V, Joseph V, Basu S, et al. Efficacy of fibrin sealant as waterproof cover in improving outcome in hypospadias surgery. African Journal of Paediatric Surgery. 2021 Oct 1;18(4):215–8. https://doi.org/10.4103/AJPS.AJPS_132_20

32. Thomas DT, Karadeniz Cerit K, Yener S, Kandirici A, Dagli TE, Tugtepe H. The effect of dorsal dartos flaps on complication rates in hypospadias repair: A randomised prospective study. J Pediatr Urol. 2015 Feb 1;11(1):23.e1-23.e4. https://doi.org/10.1016/J.JPUROL.2014.07.010
